# Evaluating Antigen- and Vector-Specific Immune Responses of a Recombinant Pichinde Virus-Based Vaccine Expressing the Lymphocytic Choriomeningitis Virus Nucleoprotein

**DOI:** 10.3390/vaccines12121450

**Published:** 2024-12-23

**Authors:** Michaela Cain, Qinfeng Huang, Shania Sanchez, Hinh Ly, Yuying Liang

**Affiliations:** Department of Veterinary and Biomedical Sciences, College of Veterinary Medicine, University of Minnesota, Saint Paul, MN 55108, USA; cain0166@umn.edu (M.C.); huangq@umn.edu (Q.H.);

**Keywords:** arenavirus, Pichinde virus, LCMV, vaccine, viral vector, vaccine immunity, anti-vector immunity

## Abstract

Background: Live viral vector-based vaccines are known to elicit strong immune responses, but their use can be limited by anti-vector immunity. Here, we analyzed the immunological responses of a live-attenuated recombinant Pichinde virus (PICV) vector platform (rP18tri). Methods: To evaluate anti-PICV immunity in the development of vaccine antigen-specific immune responses, we generated a rP18tri-based vaccine expressing the lymphocytic choriomeningitis virus (LCMV) nucleoprotein (NP) and administered four doses of this rP18tri-NPLCMV vaccine to mice. Using MHC-I tetramers to detect PICV NP38-45 and LCMV NP396-404 epitope-specific CD8+ T cells, we monitored vector- and vaccine-antigen-specific immune responses after each vaccination dose. Results: LCMV NP396-404-specific effector and memory CD8+ T cells were detected after the first dose and peaked after the second dose, whereas PICV NP38-45-specific memory CD8+ T cells increased with each dose. PICV-binding IgG antibodies peaked after the second dose, while anti-PICV neutralizing antibodies (NAbs) remained low even after the fourth dose. Immunization with the rP18tri-NPLCMV vaccine significantly reduced LCMV viral titers in a chronic LCMV Clone 13 infection model, demonstrating the protective role of LCMV NP-specific T cells. Conclusion: These findings provide important insights into the antigen- and vector-specific immunity of the rP18tri-NPLCMV vaccine and support the development of NP-based vaccines against arenavirus pathogens.

## 1. Introduction

Viral vector-based vaccines utilize genetically modified, weakened viruses to deliver antigens, stimulating immune responses without the need for adjuvants [1,2]. They are generally cost-effective and can be highly immunogenic [3,4]. Current viral vectors include both replication-competent and replication-incompetent platforms derived from various RNA and DNA viruses, such as adenoviruses, adeno-associated viruses (AAVs), vesicular stomatitis virus (VSV), lentiviruses, and poxviruses [e.g., Modified Vaccinia Virus Ankara (MVA) and Canarypox vector] [5]. The FDA has approved two viral vectored vaccines for human use: the Janssen adenovirus-vectored COVID-19 vaccine, which had its emergency use authorization (EUA) revoked in 2023 due to reports of blood clots post-vaccination, and ERVEBO, a recombinant VSV-vectored Ebola vaccine that has shown good protection against Ebola Zaire [6].

A major limitation of viral vectored vaccines is anti-vector immunity. Prior exposure to the vector virus or repeated vaccinations can lead to immune responses against the vector itself, rendering it less effective against vaccine antigen(s) [2,4]. While the availability of multiple serotypes and capsid variants makes AAVs an attractive vector, they have limited transgene capacity and lower immunogenicity compared to other viral vectors [4]. Poxvirus vectors can accommodate large transgene inserts and have shown high immunogenicity in preclinical studies [5,7]. Nevertheless, further research evaluating the potential impact of anti-vector immunity with MVA-vectored vaccines has shown reduced immunogenicity following multiple doses of the vaccine [8]. Additionally, highly attenuated replication-incompetent poxvirus vectors do not grow to high titers, posing challenges for large-scale production [9]. There are also safety concerns with some viral vectors: lentiviral vectors may pose a risk of insertional mutagenesis, and live-attenuated viral vectors could revert to a virulent form or cause disease in immunocompromised individuals [4]. Therefore, there is an urgent need for new viral vector platforms that can ensure both safety and efficacy while eliciting robust and long-lasting immune responses.

Our lab has developed a replication-competent Pichinde virus (PICV)-based vaccine platform (rP18tri) [10,11]. PICV is a non-pathogenic RNA virus within the Arenaviridae family. The natural host for PICV is the Colombian rice rat (*Oryzomys albigularis*). PICV has very low seroprevalence and pre-existing immunity in humans, even in the local population [12,13,14]. PICV’s genome consists of two RNA segments encoding four essential genes in an ambisense coding strategy. The large (L) RNA segment encodes the viral matrix protein (Z) and the large RNA-dependent RNA polymerase (L), whereas the small (S) segment encodes the viral glycoprotein (GPC) and nucleoprotein (NP). Using a reverse genetics strategy, we divided the S RNA segment into S1 and S2 RNA segments, each containing an essential viral gene (either PICV NP or PICV GPC) and a multiple cloning site (MCS) for the insertion of a foreign gene, resulting in a recombinant rPICV with three RNA segments (rP18tri) [10,11].

The rP18tri vector offers several unique advantages as a vaccine platform. PICV has no pre-existing immunity in the general population and shows a good safety profile [10]. PICV naturally targets antigen-presenting cells (APCs), which enhance antigen presentation on both MHC class I and class II molecules, promoting strong cellular and humoral immune responses [10,15]. Antigen-specific immune responses can be further increased by homologous boosting [10,11]. However, the potential development of anti-vector immunity and its impact on vaccine efficacy after multiple doses requires further investigation. The rP18tri vector can accommodate two extra open-reading frames (ORFs) of up to 2 kb each and has proven effective in inducing both systemic and mucosal immunity across multiple animal species via various vaccination routes [16,17,18]. Its ability to stimulate robust T cell immunity, apart from the antibody response, makes the rP18tri vector an ideal candidate for developing vaccines against pathogens for which cell-mediated immunity (CMI) plays a critical protective role, such as arenavirus pathogens [19,20,21,22].

The Arenaviridae family includes multiple significant human pathogens. Several Old World (OW) and New World (NW) arenaviruses can cause severe and lethal hemorrhagic fever (HF) diseases, for example, Lassa fever caused by the OW Lassa virus (LASV) in West Africa and Argentine hemorrhagic fever caused by the NW Junin virus (JUNV) in South America [23]. Lymphocytic choriomeningitis virus (LCMV) is an OW arenavirus with a global distribution and can cause neurological disorders, such as meningitis and encephalitis, congenital infections, and transplant-associated hemorrhage and death [24]. Currently, there are no FDA-approved antivirals or vaccines for these arenavirus pathogens. Previous research has suggested that CD8+ T cells play an important protective role against arenavirus infections [19,25], while neutralizing antibodies (NAbs) contribute to the protection against NW HF-causing arenaviruses but do not seem to play a major role against LASV or LCMV during infection or vaccination [19,22].

In this study, we generated a recombinant rP18tri-based vaccine expressing LCMV NP and assessed the development of both antigen- and vector-specific immune responses following multiple doses in mice. We observed high levels of antigen-specific effector and memory CD8+ T cells, which peaked after the second dose. While vector-specific antibodies and CD8+ T cells are highly elevated, no significant anti-vector NAbs were detected even after four homologous doses. This rP18tri-NPLCMV vaccine effectively reduced the LCMV viral load in a chronic LCMV infection model in mice. Our study establishes the optimal vaccination strategy for the rP18tri vector and demonstrates the role of NP-specific CD8+ T cells in generating protective immunity against LCMV, offering a promising approach for developing vaccines against other arenavirus pathogens.

## 2. Materials and Methods

### 2.1. Ethics Statements

The research described in this manuscript received approval from the Institutional Biosafety Committee at the University of Minnesota, Twin Cities, under the protocol ID 309-41371H. Additionally, all procedures involving animals were reviewed and approved by the Institutional Animal Care and Use Committee (IACUC) at the same institution under protocol IDs 2302-40834A and 2302-40819A.

### 2.2. Mammalian Cells, Plasmids, and Viruses

Baby hamster kidney cells (BHK21 and ATCC CCL-81, Manassas, VA, USA) and African green monkey kidney cells (Vero and ATCC CCL-10, Manassas, VA, USA) were cultured in Dulbecco’s modified eagle medium (DMEM) (Fisher Scientific) supplemented with 10% fetal bovine serum (FBS) (Sigma Aldrich, St. Louis, MI, USA) and 100 U/mL penicillin-streptomycin (P/S; Gibco, Gand Island, NE, USA). BSRT7-5 cells, provided by K.K. Conzelmann (Ludwig-Maximilians-Universität, Germany), are part of a BHK-21 derivative line that stably expresses T7 RNA polymerase. These cells were maintained in minimal essential medium (MEM) (Invitrogen-LifeTechnologies, Waltham, MA, USA) containing 10% FBS, 1000 μg/mL Geneticin (Invitrogen-LifeTechnologies, MA, Waltham, USA), and 100 U/mL penicillin-streptomycin (P/S; Gibco).

Three plasmids were used to generate rP18tri-NPLCMV: (1) pP18S1-GPC/MCS, encoding PICV GPC and a multiple cloning site (MCS) to clone the gene of interest; (2) pP18S2-MCS/NP, encoding PICV NP and an MCS; and (3) pP18L plasmid (the L plasmid), which produces the entire L RNA segment and does not contain an MCS.

Recombinant rP18tri vector and rP18tri-vectored vaccines were obtained by the reverse genetics strategy described previously [10,11]. Wild-type PICV (P18 strain) was described in a previous publication [26]. LCMV Clone 13 (cl13 strain) was obtained from Dr. Viava Vezys at the University of Minnesota. All viruses were amplified in BHK-21 cells, and viral plaque assay was used to determine virus concentrations.

### 2.3. Tetramers and Antibodies

Phycoerythrin (PE)-conjugated MHC-I tetramers, H-2Db LCMV NP396-404 (FQPQNGQFI), and H-2K(b) PICV NP38-45 (SALDFHKV) were sourced from the NIH Tetramer Core Facility at Emory University. Antibodies and reagents used for T cell analysis of immunized mice were purchased from commercial sources, including Purified Rat Anti-Mouse CD16/CD32 Fc Block (BD Biosciences, Franklin Lakes, NJ, USA), allophycocyanin (APC)-labeled anti-CD3 (17A2) (Biolegend, San Diego, CA, USA), brilliant violet (BV) 605-labeled CD62L (BD Horizon, Franklin Lakes, USA), BV510-labeled anti-CD44 (Biolegend, San Diego, USA), fluorescein isothiocyanate (FITC)-labeled anti-CD4 (Biolegend, San Diego, USA), and peridinin chlorophyll protein (PerCP)-Cy5.5-labeled anti-CD8 (53-6.7) (eBioscience, San Diego, CA, USA). Fixable viability Ghost Dye Red 710 (Cytek, Fremont, USA) was used to differentiate between live and dead cells.

### 2.4. UV Inactivation of LCMV and rPICV Virions

LCMV Clone 13 (cl13 strain) or rPICV (rP18 strain) virus was cultured in BHK21 cells for 48 h, and the supernatant was collected into 50 mL conical tubes (SARSTEDT) and cleared of debris by centrifugation at 2000× *g* for 15 min at 4 °C in the Sorvall ST 16R refrigerated centrifuge (Thermo Fisher Scientific, Waltham, MA, USA). The supernatant was collected and concentrated by ultracentrifugation through 8% OptiPrep/sodium chloride-tris-EDTA buffer solution (STE) at 27,000 rpm for 2 h at 4 °C in a SW28 rotor in a Beckman Coulter Optima L-90K Ultracentrifuge. The viral pellet was resuspended in sterile PBS for 12 h at 4 °C. UV inactivation was conducted by UV exposure at 254/366 nm for 30 m (Entela Certified model UVGL-58), and viral inactivation was confirmed by plaque assay. The protein concentration of inactivated viruses was quantified using the Pierce BCA Protein Assay kit (Thermo Fisher Scientific, Waltham, MA, USA).

### 2.5. Generation of Recombinant rP18tri Vaccine Encoding LCMN NP Gene (rP18tri-NPLCMV)

**Figure 1:**
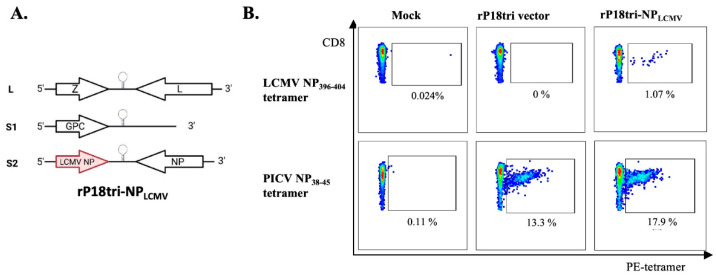
The rP18tri-NPLCMV vaccine elicited PICV and LCMV NP-specific CD8+ T cells. (**A**) The genomic organization of the tri-segmented rP18tri-NPLCMV virus with the LCMV NP gene inserted into the S2 segment in the positive orientation. (**B**) Representative flow plots of LCMV NP396-404 (**top panels**) and PICV NP38-45 (**bottom panels**) MHC-I tetramer staining for PBMCs from mice immunized with PBS (mock), the rP18tri vector, or rP18tri-NPLCMV.

The rP18tri-based vaccine was generated as described previously [10,11]. The protein sequence of LCMV NP (NC_077807.1) was obtained from GenBank. DNA fragments encompassing the full-length LCMV NP gene were codon-optimized for mammalian cell expression and chemically synthesized by Genewiz (Azenta Life Sciences, Waltham, MA, USA). The DNA fragment encoding the LCMV gene was cloned into the S2 vector (Figure 1A) [10,11]. The resulting plasmid was verified by sequencing.

To rescue recombinant rP18tri-NPLCMV, BSRT7-5 cells, seeded at a density of 1 × 10^5^ cells per mL in a 6-well plate, were transfected with three plasmids encoding the PICV L segment and S1 and S2 segments using Lipofectamine™ 3000 Transfection Reagent (Thermo Fisher Scientific, L3000015, Waltham, MA, USA). Supernatants from these cells were harvested and used for plaque assays on Vero cells. Individual plaques were isolated to infect BHK-21 cells grown in 10 cm^2^ plates for 72 h to generate viral vaccine stocks.

Viral RNA was extracted from the vaccine stocks using the QIAamp Viral RNA Kit (Qiagen, Germantown, MD, USA) and subjected to RT-PCR for full-genome amplification and sequencing to verify the identity of the vaccine.

### 2.6. Viral Plaque Assay

Plaque assay was conducted following the established procedure to quantify the infectious dose of recombinant rP18tri-NPLCMV, rPICV, and rLCMV [26]. Vero cells were used to carry out seeding into 6-well plates at 60–70% confluency (4 × 10^5^ cells/well). Cells were infected the next day with 0.5 mL of serially diluted virus in PBS for 1 h at 37 °C. After washing with PBS, the infection medium was removed, and cells were incubated in fresh MEM supplemented with 0.4% agar and 10% FBS. Cells were cultivated for 5 to 7 days at 37 °C and 5.0% CO_2_. A neutral red solution (1:50) in 0.5% agar-MEM-10% FBS was used to stain virus plaques.

### 2.7. Mouse Immunizations, Infection, and Tissue Collection

C57BL/6 mice (n = 6) were immunized intraperitoneally (IP) with either phosphate-buffered saline (PBS) or 1 × 10^5^ PFU of the rP18tri vector or the rP18tri-NPLCMV vaccine for a total of four doses with an interval of 45 days. Following vaccination, blood was collected into lithium heparin tubes (Greiner Bio-One, Monroe, LA, USA) at 7, 30, and 45 d post-vaccination. Peripheral blood mononuclear cells (PBMCs) were isolated by lysing red blood cells (RBCs) with 1X RBC lysis buffer (eBiosciences, San Diego, CA, USA), followed by washing the cell pellet with PBS containing 2% fetal bovine serum (FBS) (Sigma Aldrich, St Louis, CA, USA). PBMCs were then used for CD8+ T cell evaluation by MHC-I tetramer analysis. At 14 and 37 d after each dose, blood was collected in 1.7 mL tubes (Greiner Bio-One) to prepare serum samples for evaluation of antibody responses by ELISA and neutralization assay.

For challenge studies, C57BL/6 mice (n = 4) were immunized intraperitoneally (IP) with either PBS, rP18tri, or rP18tri-NPLCMV vaccine at 1 × 10^5^ PFU in a prime-boost regimen at a 30 d interval. At 42 d post-boost, mice were challenged retro-orbitally with 3 × 10^6^ PFU of LCMV Clone13 (cl13 strain). Mice were weighed daily for 14 d before euthanization. Blood, kidneys, and livers were collected in 1 mL of DNA/RNA Shield (Zymo Research, Irvine, CA, USA) for viral RNA quantification via RT-qPCR.

### 2.8. LCMV RT-qPCR

Total RNAs were extracted from tissues using Zymo Research Quick-DNA/RNA Viral Magbead Extraction Kit and quantified for LCMV RNA levels with Luna Universal One-step RT-qPCR Kit (New England Biolabs, Ipswhich, MA, USA) using LCMV NP-specific primers (GCCATAGTTAGACTTGGCAT and CCTACAGATAGTTGGGATGAG) in a Bio-Rad CFX Optics Module. RT-qPCR was performed using a Bio-Rad T100 Thermal Cycler at 48 °C for 30 m, 94 °C for 1 m, 94 °C for 15 s, 60 °C for 30 s, and 68 °C for 1 m for 40 cycles, and then at 68 °C for 5 m. Viral RNA concentration was determined based on a standard curve.

### 2.9. Evaluation of Antigen-Specific CD8+ T Cells by MHC-I Tetramer Analysis

Isolated PBMCs were incubated with PE-labeled tetramers, fixable viability stain Ghost Dye Red 710, APC-labeled anti-CD3, PerCP-Cy5.5-labeled anti-CD8, FITC-labeled anti-CD4, BV510-labeled anti-CD44, BV605-labeled CD62L, and Purified Rat Anti-Mouse CD16/CD32 Fc Block for 45 m on ice. Cells were then washed three times with PBS/2% FBS. Sample acquisition was performed on a BD FACSCelesta Flow Cytometer, and data analysis was performed using FlowJo (10.9).

### 2.10. Enzyme-Linked Immunosorbent Assay (ELISA)

The levels of anti-PICV IgG antibodies in mouse serum were determined by ELISA. Briefly, 96-well ELISA plates (NUNC) were coated with inactivated PICV virions (100 ng/well), washed with PBST 0.05% (Sigma-Aldrich), and incubated with heat-inactivated serially diluted serum for 1 h at 37 °C. After washing with PBS + 0.05% Tween20, HRP-conjugated anti-mouse IgG antibody (1:1000, R&D Systems, Minneapolis, MN, USA) was added to the plates and incubated for 1 h at 37 °C. The plates were washed with PBS + 0.05% Tween20 and incubated with ABTS substrate and stop solution (Roche), and absorbance was measured at 450 nm. The end-point IgG titer was calculated using the four-parameter logistic (4-PL) fitting model in Prism, with the cut-off value defined as mean + 4× standard deviations of control wells.

### 2.11. PICV Neutralization Assays

Vero cells were seeded into a flat-bottom 96-well plate (NUNC) at 2 × 10^4^ cells per well and incubated overnight at 37 °C with 5% CO_2_. The next day, mouse serum samples were heat-inactivated at 56 °C for 30 min, serially diluted two-fold, and incubated with a fixed dose of recombinant PICV luciferase (LUC) reporter virus (rP18tri-LUC) at 37 °C for 1 h. The virus–serum mixture was added to the plate and incubated at 37 °C for 24 h. Cells were washed once with PBS and lysed with 50 µL/well of 1X Luciferase Cell Culture Lysis Reagent (Promega, catalog number E1531, Madison, WI, USA). Firefly LUC activity in lysates was measured using the Fluc luciferase Assay System (Promega, Madison, WI, USA). The neutralizing antibody (NAb) titer, defined as the serum dilution that reduced the LUC activity by 50%, was quantified by non-linear regression in Prism 10 (GraphPad, San Diego, CA, USA).

### 2.12. Statistical Analysis

All statistical analyses were performed using GraphPad Prism 10.0.2. Antibody titers were log10-transformed and analyzed using Sigmoidal 4-parameter. For statistical analysis of LCMV NP tetramer staining between groups of the same time point, unpaired two-tailed *t*-tests with Welch’s correction and a 95% confidence interval were performed. For analysis of LCMV NP tetramers of the same group at different time points, paired two-tailed *t*-tests were performed with a confidence interval of 95%. *p*-values of less than 0.05 were considered significant, and *p* values of less than 0.01 were considered highly significant.

## 3. Results

### 3.1. Detection of Antigen- and Vector-Specific CD8+ T Cells by Tetramer Staining in Mice Immunized with rP18tri-NPLCMV Vaccine

LCMV NP-specific CD8+ T cells can be conveniently evaluated by a well-established MHC-I tetramer assay with the H-2Db LCMV NP396-404 tetramer [27,28,29]. We thus used LCMV NP as a model T cell antigen and generated a rP18tri-vectored vaccine rP18tri-NPLCMV encoding LCMV NP (Figure 1A).

To determine whether rP18tri-NPLCMV can elicit LCMV NP antigen-specific CD8+ T cells, we immunized (IP) mice with PBS (mock), rP18tri vector, or rP18tri-NPLCMV. PBMCs were analyzed 7 d post-vaccination using MHC-I tetramer staining. LCMV NP396-404 tetramer-positive CD8+ T cells were detected only in mice immunized with rP18tri-NPLCMV but not with the mock or the rP18tri vector, while PICV NP38-45 tetramer-positive CD8+ T cells were present at high levels in mice immunized with both the vector and rP18tri-NPLCMV but were undetected in the mock group (Figure 1B). The LCMV NP396-404 epitope is FQPQNGQFI, and the corresponding sequence in PICV NP is YQPDTGNYI, whereas the PICV NP38-45 epitope is SALDFHKV, and the corresponding sequence in LCMV NP is NGLDFSEV. Thus, even though LCMV and PICV NP proteins show 79% similarity and 50% identity scores, the two MHC-I tetramers do not cross-react with CD8+ T cells induced by the opposing NP antigen. Our data demonstrate that rP18tri-NPLCMV can elicit strong antigen- and vector-specific T cell responses, which can be specifically detected by the two MHC-I tetramers.

### 3.2. Evaluation of Antigen-Specific Effector CD8+ T Cells After Multiple-Dose Immunization

**Figure 2:**
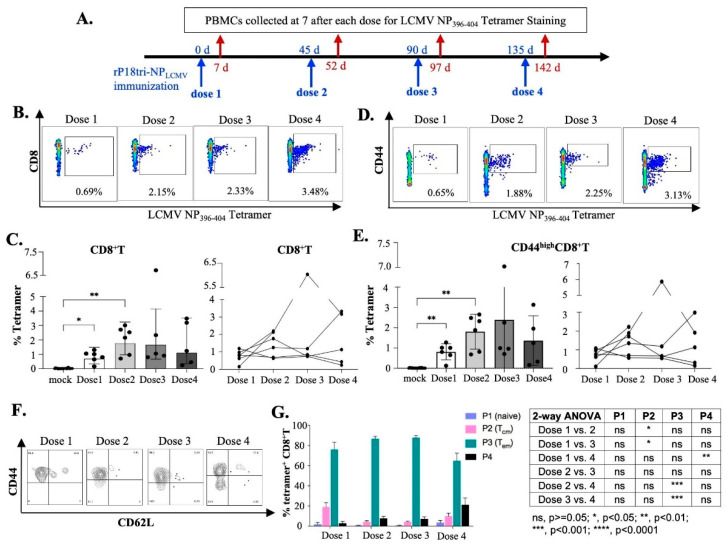
Evaluation of LCMV NP396-404 epitope-specific effector CD8+ T cells elicited 7 d after each dose of rP18tri-NPLCMV immunization. (**A**) Experimental procedure. Mice (n = 6) were immunized (IP) with rP18tri-NPLCMV for 4 doses (shown in blue arrows) with 45 d interval. PBMCs collected 7 d after each dose (red arrows) were incubated with PE-labeled MHC-I LCMV NP396-404 tetramer, along with antibodies against surface markers (CD3, CD4, CD8, CD44, and CD62L), and analyzed by flow cytometry. (**B**) Representative flow cytometry plots of tetramer-positive effector CD8+ T cells 7 d after each dose of vaccination. (**C**) Percentage of tetramer-positive effector CD8+ T cells 7 d after each dose shown for group of immunized mice (**left**) and for individual mice (**right**). (**D**) Representative flow cytometry plots of tetramer-positive CD44highCD8+ T cells 7 d after each dose of vaccination. (**E**) Percentage of tetramer-positive CD44highCD8+ T cells 7 d after each dose shown for group of immunized mice (**left**) and for individual mice (**right**). (**F**) Representative flow cytometry plots of tetramer-positive CD8+ T cells by CD44 and CD62L gating. (**G**) Percentage of four populations by CD44 and CD62L gating of tetramer-positive CD8+ T cells: CD44lowCD62Lhigh denoted as P1 (naive) cells, CD44highCD62Lhigh as P2 (TCM), CD44highCD62Llow as P3 (TEM), and CD44lowCD62Llow as P4. Table shows statistical significance of comparison between two doses for each cell population. Significance values: ns, *p* ≥ 0.05; *, *p* < 0.05; **, *p* < 0.01; ***, *p* < 0.001; ****, *p* < 0.0001.

To evaluate the changes in antigen-specific CD8+ T cells elicited after repeated exposure to the viral vectored vaccine, we immunized (IP) mice with PBS (mock) or rP18tri-NPLCMV for a total of four doses with a 45 d interval and collected PBMCs 7 d after each dose for LCMV NP396-404 tetramer staining (Figure 2A). The interval time was selected based on a previous study with another arenavirus viral vector, showing that boosting at an interval of 4 weeks or longer enhances the magnitude of the immune response [30].

The population of LCMV NP396-404 tetramer-positive CD8+ T cells elicited 7 d after each dose is shown in a representative flow cytometry image (Figure 2B), while the percentage of the tetramer-positive population over the CD8+ T cells after each dose is shown as the average of the group (Figure 2C) or for the individual mouse (Figure 2D). A significant number of LCMV NP396-404-specific CD8+ effector T cells were detected after the first dose in all vaccine-immunized mice and increased markedly after the second dose. While NP antigen-specific CD8+ T cells continued to increase after the third or the fourth dose in two mice, they remained at similar levels in two other mice and decreased in one mouse; as such, their average magnitude as a group did not increase further after the third and fourth doses of vaccination. Most of these NP tetramer-specific CD8+ T cells expressed the activation and memory T cell marker CD44high (Figure 2E). As such, the magnitude of LCMV NP396-404-specific CD44highCD8+ T cells showed a similar trend of changes after each dose of vaccination (Figure 2F,G) as that of CD8+ T cells (Figure 2C,D). These NP396-404-specific CD8+ T cells were divided into four populations by CD44 and CD62L gating, with CD44lowCD62Lhigh denoted as P1 (naive) cells, CD44highCD62Lhigh as P2 central memory cells (TCM), CD44highCD62Llow as P3 effector memory cells (TEM), and CD44lowCD62Llow as P4 [31]. These NP antigen-specific effector CD8+ T cells at 7 dpi after each dose were predominantly of the TEM phenotype (Figure 2G [31]. These data suggest that the rP18tri-NPLCMV vaccine activates antigen-specific CD8+ effector T cells, which increase significantly after a boost immunization.

### 3.3. Evaluation of Antigen-Specific Memory CD8+ T Cells After Repeated Vaccination Doses

**Figure 3:**
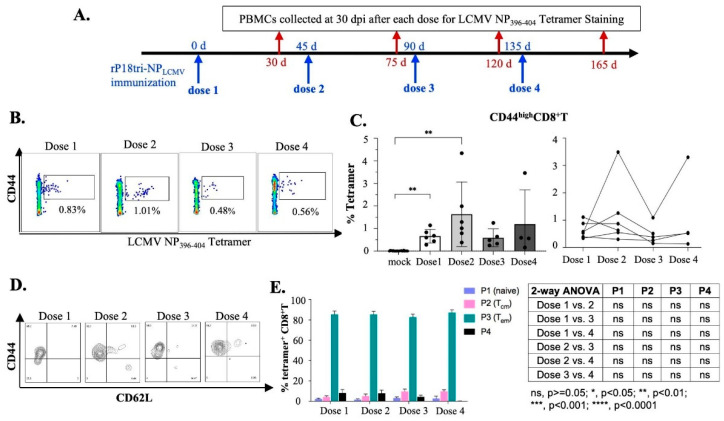
Evaluation of LCMV NP396-404 epitope-specific memory CD8+ T cells elicited 30 d after each dose of rP18tri-NPLCMV immunization. (**A**) Experimental procedure: Mice were immunized as described in Figure 2A legends. PBMCs collected at 30 d after each dose (shown in red arrows) were incubated with PE-labeled MHC-I LCMV NP396-404 tetramer, along with antibodies against surface markers (CD3, CD4, CD8, CD44, and CD62L), and analyzed by flow cytometry. (**B**) Representative flow cytometry plots of tetramer-positive CD44highCD8+ memory T cells 30 d after each dose of vaccination. (**C**) Percentage of tetramer-positive CD44highCD8+ memory T cells at 30 d after each dose is shown for group of immunized mice (**left**) and for individual mice (**right**). (**D**) Representative flow cytometry plots of tetramer-positive CD8+ T cells by CD44 and CD62L gating. (**E**) Percentage of four populations by CD44 and CD62L gating of tetramer-positive CD8+ T cells: CD44lowCD62Lhigh denoted as P1 (naive) cells, CD44highCD62Lhigh as P2 (TCM), CD44highCD62Llow as P3 (TEM), and CD44lowCD62Llow as P4. Table shows statistical significance of comparison between two doses for each cell population. Significance values: ns, *p* ≥ 0.05; *, *p* < 0.05; **, *p* < 0.01; ***, *p* < 0.001; ****, *p* < 0.0001.

To investigate whether memory CD8+ T cells developed after the vaccination, we collected PBMCs 30 d post-immunization, when effector T cells contracted following each dose, and analyzed them using LCMV NP396-404 tetramer staining with memory CD8+ T cell markers CD44highCD8+ (Figure 3A). A significant number of NP antigen-specific memory CD8+ T cells were generated after each vaccination (Figure 3B), with the highest level on average being detected after the second dose (Figure 3C left) and with relatively large variations being observed in individual mice (Figure 3C right). These NP396-404-specific memory CD8+ T cells were analyzed by CD44 and CD62L gating (Figure 3D) and were predominantly of the TEM phenotype after each vaccination [31] (Figure 3E).

### 3.4. Evaluation of Vector-Specific CD8+ T Cells After Repeated Vaccinations

**Figure 4:**
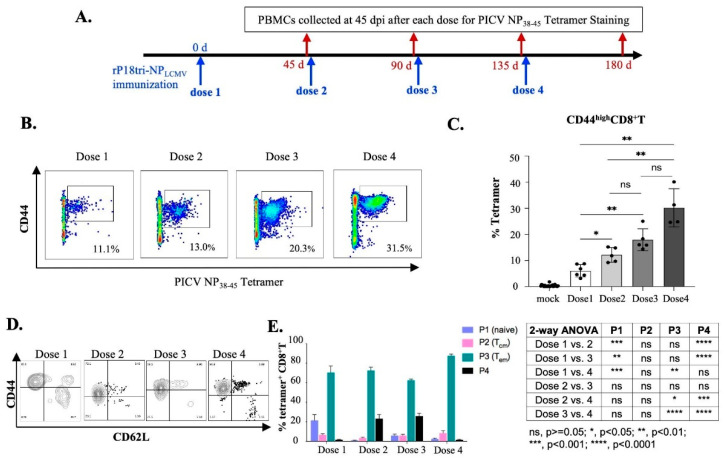
Evaluation of PICV NP38-45 epitope-specific memory CD8+ T cells elicited 45 d after each dose of rP18tri-NPLCMV immunization. (**A**) Experimental procedure: Mice were immunized as described in Figure 2A legends. PBMCs collected at 45 d after each dose (shown in red arrows) were incubated with PE-labeled MHC-I PICV NP38-45 tetramer, along with antibodies against surface markers (CD3, CD4, CD8, CD44, and CD62L), and analyzed by flow cytometry. (**B**) Representative flow cytometry plots of tetramer-positive CD44highCD8+ memory T cells 45 d after each dose of vaccination. (**C**) Percentage of tetramer-positive CD44highCD8+ memory T cells 30 d after each dose shown for group of immunized mice (**left**) and for individual mice (**right**). (**D**) Representative flow cytometry plots of tetramer-positive CD8+ T cells by CD44 and CD62L gating. (**E**) Percentage of four populations by CD44 and CD62L gating of tetramer-positive CD8+ T cells: CD44lowCD62Lhigh denoted as P1 (naive) cells, CD44highCD62Lhigh as P2 (TCM), CD44highCD62Llow as P3 (TEM), and CD44lowCD62Llow as P4. Table shows statistical significance of comparison between two doses for each cell population. ns, *p* ≥ 0.05; *, *p* < 0.05; **, *p* < 0.01; ***, *p* < 0.001; ****, *p* < 0.0001.

To assess the development of anti-vector T cell responses after repeated vaccinations, mouse PBMCs were collected 45 d post-immunization and analyzed by PICV NP38-45 tetramer staining (Figure 4A). PICV NP38-45 tetramer-specific memory CD8+ T cells were detected at high levels (Figure 4B) and increased with each dose, with statistical significance being observed between the first and second doses (Figure 4C). Using CD44 and CD62L gating, the major population of PICV NP38-45-specific memory CD8+ T cells was TEM (CD44highCD62Lhigh) after each dose (Figure 4D,E). However, P1 naive cells (CD44lowCD62Lhigh) took up 20% of the T cell population after the first dose, while P4 (CD44lowCD62Llow) reached ~ 25% after the second and third doses, which was different from LCMV NP396-404-specific memory CD8+ T cells (Figure 3E) in which populations of P1 and P4 cells were relatively rare. The notable difference between these two viral NP epitope-specific memory CD8+ T cells needs to be further investigated.

### 3.5. Evaluation of Vector-Specific Antibodies After Repeated Vaccination

**Figure 5:**
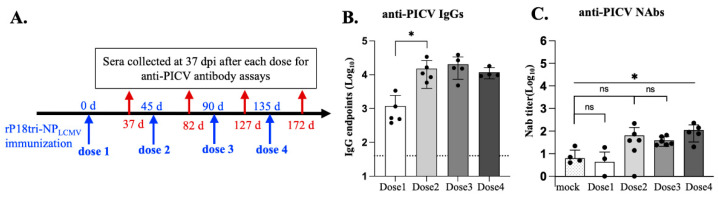
Evaluation of anti-PICV (vector) antibodies after each dose of rP18tri-NPLCMV immunization. (**A**) Experimental procedure: Mice were immunized as described in Figure 2A legends. Serum samples collected 45 d after each dose were evaluated for anti-PICV antibodies. (**B**) Anti-PICV IgGs were measured by ELISA with plates coated with inactivated rPICV virion particles and shown as endpoints in log scale. (**C**) Anti-PICV NAbs were evaluated by neutralization assay and shown as NAb titers in log scale. ns, no statistical significance. *, *p* < 0.05.

To quantify the level of PICV-binding IgGs, we collected serum samples 37 d post-vaccination after each vaccination dose and performed PICV IgG ELISA. Mice vaccinated with rP18tri-NPLCMV produced a relatively high level of PICV-specific IgGs, which increased markedly after the second dose of vaccination and remained at a similarly high level after the third and fourth doses (Figure 5B).

To determine whether these vector-specific IgGs have anti-PICV neutralizing activity, we performed a neutralization assay. The quantified neutralizing antibody (NAb) titers were not statistically significant between the mock and vaccinated groups after the first three doses, suggesting the lack of detectable neutralizing activity (Figure 5C). While the fourth dose of vaccination resulted in a statistically significant increase in the NAb titers compared to the mock group, the average titer remained low (<1 × 10^2^) (Figure 5C. This finding is consistent with the findings reported in previous publications [32,33,34] and further demonstrates that neither PICV nor the rP18tri vector elicits a detectable level of NAbs even after repeated exposures.

### 3.6. The rP18tri-NPLCMV Vaccine Reduced the LCMV Load in a Mouse Model of Chronic Infection

**Figure 6:**
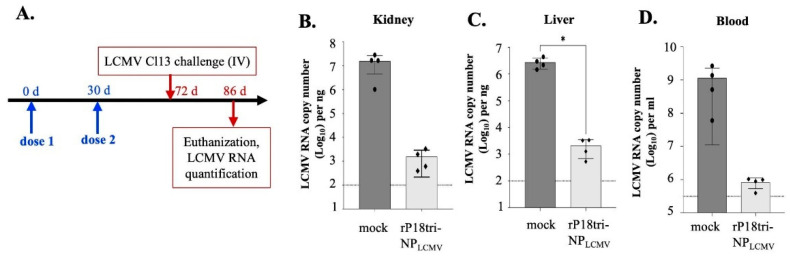
rP18tri-NPLCMV reduced LCMV viral load in mouse model of chronic infection. (**A**) Experimental procedure: Mice were immunized with PBS (mock) or rP18tri-NPLCMV for 2 doses with 30 d interval, and 42 d after second dose, they were challenged (IV) with 3 × 10^6^ PFU of LCMV cl13 strain. LCMV viral RNAs in tissues 14 d post-challenge were quantified by RT-qPCR and are shown in log scale in liver (**B**), kidney (**C**), and blood (**D**). Dotted line represents baseline for RT-qPCR detection. *, *p* < 0.05.

To determine whether LCMV NP-specific immunity confers protection, we evaluated the vaccine efficacy in the mouse model of chronic LCMV Cl13 infection [35]. C57BL/6 mice (n = 4) were immunized (IP) with PBS or rP18tri-NPLCMV using a prime-boost vaccination regimen and, at 42 d post-boost, were challenged with LCMV Cl13. The mock-immunized mice showed a relatively high LCMV viral load by RT-qPCR in the kidney, liver, and blood samples 14 d post-challenge, whereas the viral load in the rP18tri-NPLCMV-vaccinated mice decreased by 3–5 logs and was barely above the detection limit (Figure 6). These data demonstrate the functional importance of LCMV NP-specific immunity in controlling viral infection in animals.

## 4. Discussion

In this study, we conducted a comprehensive evaluation of antigen- and vector-specific CD8+ T cells, along with anti-vector antibody responses, throughout the multiple-dose immunization of mice with rP18tri-NPLCMV, a replication-competent rP18tri viral vectored vaccine expressing LCMV NP as a model T cell antigen. We show that rP18tri-NPLCMV elicits strong antigen-specific effector and memory cytotoxic T lymphocytes (CTLs), as evidenced by high levels of LCMV NP396-404 epitope-specific CD8+ and CD44high CD8+ T cells during the expansion (7 d) phase and after the contraction (30 d) period. The antigen-specific T cell response was robust following the first dose and became even stronger after the second dose. A strong vector-specific CTL response was also detected, as shown by a large number of PICV NP38-45 CTLs that increased after the administration of multiple doses. Despite the high level of anti-PICV IgGs, particularly after the second dose, neutralizing activity against PICV remained undetected or at very low levels even after the fourth dose. Together, these findings suggest that although a single dose of the replication-competent rP18tri vectored vaccine can elicit a strong antigen-specific CTL response, which may already provide sufficient protection, a boost dose significantly enhances vaccine immunity. Thus, a prime-boost vaccination strategy can be used with rP18tri-vectored vaccines to achieve the strongest vaccine immunity and protection, which is especially relevant for vaccine development against complex diseases such as cancers, tuberculosis, and malaria.

Our data showing the peak of antigen-specific memory CD8+ T cells after the second dose of rP18tri-NPLCMV are consistent with a previous study using two arenavirus vectors, rPICV and rLCMV [36], where the highest immune responses were observed after the second dose of homologous boosting. The exact mechanism for the increased antigen-specific memory CD8+ T cells after prime-boost immunization is unclear, but it may result from a robust recall (memory) response, with a much higher number of effector T cells generated during the expansion phase. PICV (vector)-specific immunity also seems to peak after the second dose of vaccination, as observed in the anti-PICV binding IgGs in this study (Figure 5B) and PICV-specific T cells in a prior study [37]. Although the number of PICV NP38-45 tetramer-positive memory CD8+ T cells continued to increase after the third and fourth doses (Figure 4C), they targeted a highly immunodominant epitope, while the total PICV-specific T cells, as evaluated by ELISPOT, did not increase further after the second dose [37]. The high levels of vector-specific CD8+ T cells after the second dose may limit the further increase in antigen-specific T cells after additional doses. To mitigate anti-vector immunity developed after multiple doses, a possible solution is to use heterologous prime-boost strategies. Previous studies have shown that heterologous boosting with different arenavirus vectors, such as alternating between rPICV and rLCMV, can bypass vector-specific immunity and refocus the immune response on the target antigen [38]. This approach has already shown success in early-phase human clinical trials for HPV16+ cancer vaccines, where it induced strong tumor-specific CD8+ T cell responses and long-term immunity [36,39].

Although high titers of PICV-specific IgG antibodies were generated, particularly after the second dose, anti-PICV NAbs were not observed until the fourth dose, and even then, they were only present at a low titer (Figure 5B,C). Our finding is consistent with previous reports showing that the extensive post-translational glycosylation of the PICV envelope glycoprotein, which forms “glycan shields”, can limit the development of NAbs against the virus [32,33,34]. Since using anti-vector NAbs is a major mechanism to reduce the efficacy of viral vectored vaccines, the lack of anti-PICV NAbs despite repeated exposures represents a key advantage for the rP18tri vaccine vector. It remains to be determined whether these non-neutralizing PICV-specific IgGs could influence antigen immunity through Fc effector functions.

Developing effective vaccines for arenaviruses is challenging due to the high genetic diversity of viral strains and limited knowledge of the protective antigen(s) and protective immunity. Most if not all of the experimental arenavirus vaccines based on viral vectors and RNA/DNA platforms utilize the full-length glycoprotein GPC as the vaccine antigen [23]. However, GPC has the highest sequence diversity among the arenaviral proteins, and studies have indicated that vaccines containing only one strain of GPC failed to provide adequate protection against other arenavirus strains [40,41]. Arenaviral NP is an attractive vaccine target because it is abundantly expressed in arenavirus-infected cells, is more conserved in sequence across arenavirus strains, and is highly immunogenic [42]. Notably, NP contains both CD4+ and CD8+ T cell epitopes, some of which are conserved among arenaviruses [43,44]. Previous studies have suggested that CD4+ T cell responses targeting LASV NP play an important role in the early control of LASV infection [21,45]. Our study demonstrates the protective role of LCMV NP-specific immune responses, resulting in a significant reduction in viral titers in various tissues in a chronic LCMV infection model (Figure 6). These anti-LCMV-NP immune responses likely control viral infection by inducing a robust memory T cell response and promoting enhanced T cell differentiation and function. In summary, our study characterizes the development of vector- and antigen-specific immune responses during multi-dose immunization with the rP18tri-NPLCMV vaccine. It establishes the prime-boost vaccination strategy for the rP18tri-based vaccine platform to induce the strongest vaccine immunity and provides evidence for the protective role of NP-specific immunity against arenavirus pathogens. These findings advance our understanding of the rP18tri vaccine vector platform and demonstrate its potential for developing vaccines against various infectious diseases, including those caused by arenavirus pathogens.

## 5. Patents

The rP18tri vector described in this manuscript is the subject of an approved patent, No. PCT/US2015/051337.

## Data Availability

The original contributions presented in this study are included in the article/Supplementary Material. Further inquiries can be directed to the corresponding authors.

## Supplementary Materials

The following supporting information can be downloaded at https://www.mdpi.com/article/10.3390/vaccines12121450/s1, Figure S1: Flow cytometry gating strategy.
