# Supplementary figures and images for: Evaluating Antigen- and Vector-Specific Immune Responses of a Recombinant Pichinde Virus-Based Vaccine Expressing the Lymphocytic Choriomeningitis Virus Nucleoprotein
Source: Vaccines (Basel). 2024 Dec 23;12(12):1450. doi: 10.3390/vaccines12121450 (PMC11680116; doi:10.3390/vaccines12121450)

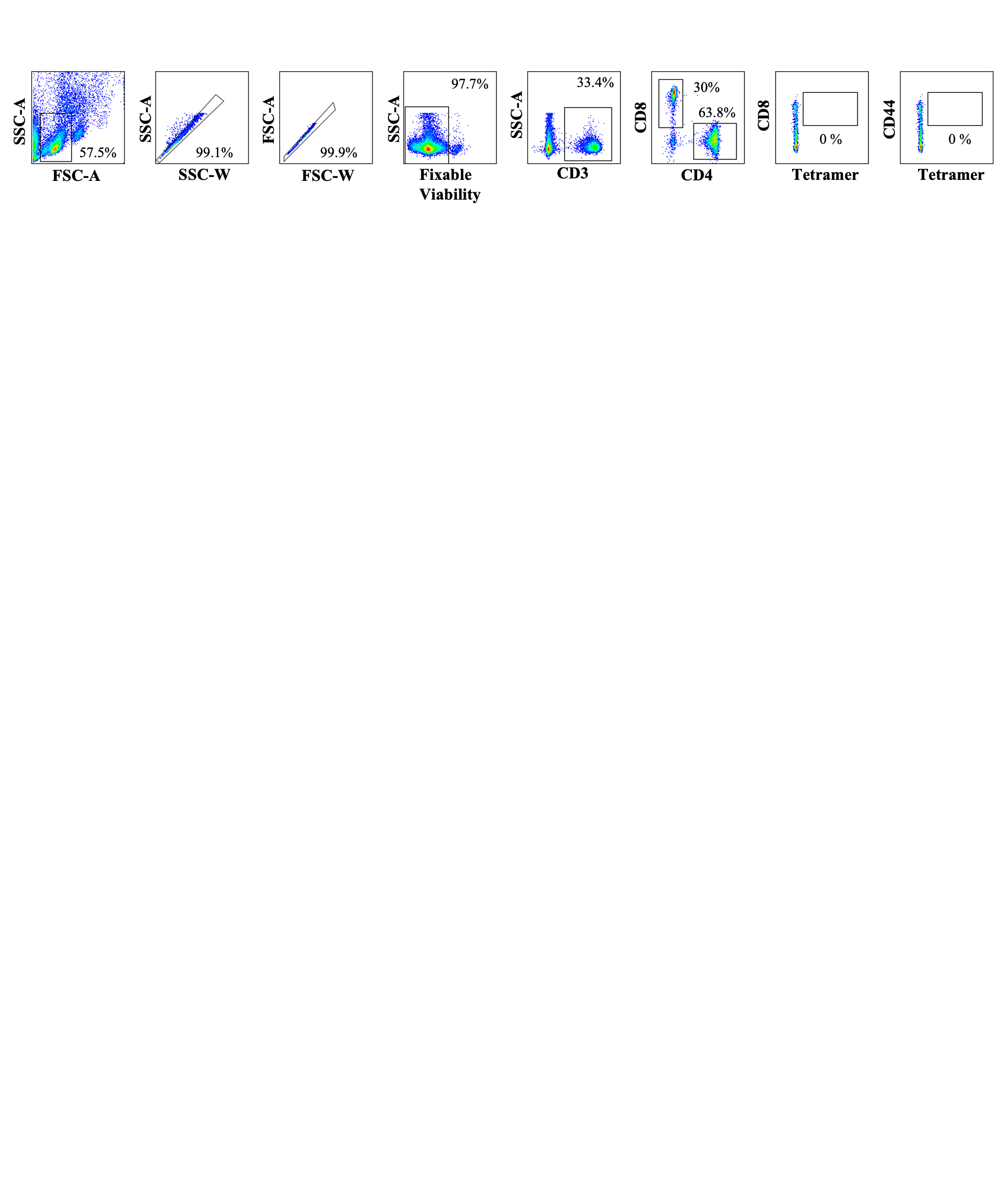

Supplement: Supplementary file 1 [file vaccines-12-01450-s001.zip › vaccines-3340064-supplementary.tiff]
